# Pre-hospital THRIVE score predicts the thrombolysis in cerebral infarction outcome post endovascular thrombectomy: an emergency medical service study

**DOI:** 10.1186/s12873-025-01352-3

**Published:** 2025-09-29

**Authors:** Hui-An Lin, Sheng-Feng Lin, Chyi-Huey Bai

**Affiliations:** 1 School of Public Health, College of Public Health, Taipei Medical University, Taipei, Taiwan; 2 Department of Emergency Medicine, School of Medicine, College of Medicine, Taipei Medical University, Taipei, Taiwan; 3 Department of Emergency Medicine, Taipei Medical University Hospital, Taipei, Taiwan; 4 Department of Public Health, School of Medicine, College of Medicine, Taipei Medical University, Taipei, Taiwan; 5 Department of Evidence-Based Medicine, Taipei Medical University Hospital, Taipei, Taiwan; 6 Department of Medical Research, Taipei Medical University Hospital, Taipei, Taiwan; 7 Department of Public Health, School of Medicine, College of Medicine, Taipei Medical University, No. 250, Wuxing St., Xinyi Dist, Taipei City, 110 Taiwan

**Keywords:** Acute ischemic stroke, Thrombectomy, THRIVE score, Recanalization

## Abstract

**Background:**

The Totaled Health Risks in Vascular Events (THRIVE) score, which ranges from 0 to 9, incorporates factors such as age, the National Institutes of Health Stroke Scale (NIHSS), and the presence of comorbidities including atrial fibrillation, diabetes mellitus, and hypertension. This study aimed to evaluate the predictive value of the THRIVE score on immediate revascularization status following endovascular thrombectomy (IAT).

**Methods:**

This retrospective cohort study utilized data from the ASSST dataset, covering the period from January 1, 2017, to May 31, 2022. Patients with acute ischemic stroke who underwent IAT were included in this analysis. The association between the THRIVE score and recanalization status—assessed using the Thrombosis in Cerebral Ischemia (TICI) scale (grades 2b/3)—was evaluated employing logistic regression models.

**Results:**

A total of 485 participants who received IAT were included in the analysis. Our findings revealed that a lower THRIVE score (OR 1.15, 95% CI 1.01–1.30), male sex (OR 1.81, 95% CI 1.15–2.87), and IAT performed following intravenous thrombolysis (OR 1.72, 95% CI 1.08–2.74) were significantly associated with successful revascularization. Youden’s index identified a THRIVE score threshold of < 5 as optimal for predicting outcomes. Patients with a THRIVE score < 5 exhibited a higher likelihood of successful revascularization (OR 1.82, 95% CI 1.10–3.03).

**Conclusion:**

A lower THRIVE score (< 5) is associated with an increased likelihood of successful revascularization following IAT in patients with acute ischemic stroke, particularly among women.

**Supplementary Information:**

The online version contains supplementary material available at 10.1186/s12873-025-01352-3.

## Introduction

Acute ischemic stroke is a leading cause of mortality globally and significant contributor to long-term disability among survivors [1]. Nearly half of acute ischemic stroke survivors experience persistent functional impairment that impact the quality of life [2, 3]. The cornerstone of acute ischemic stroke management includes airway protection, stabilization of breathing, optimization of intravenous fluid, and revascularization therapies to restore cerebral circulation. Among these, intravenous thrombolysis (IVT) with alteplase remains an important modality for acute cerebral arterial occlusion. However, the therapeutic benefits of IVT are limited by its narrow treatment window—3 to 4.5 h since the stroke onset [4, 5] —and its reduced efficacy in treating large vessel occlusion (LVOs) [6–9]. Consequently, only a small proportion of patients can fully benefit from IVT.

The advent of a new generation device of intra-arterial thrombectomy (IAT) devices using stent retriever has substantially improved clinical outcomes and reduced adverse events associated with endovascular treatment [10–13]. In 2015, several landmark randomized control trials—MR CLEAN [14], ESCAPE [15], SWIFT PRIME [16], EXTEND-IA [17], and REVASCAT [18] trial—established the efficacy of IAT in achieving better functional outcomes with fewer vascular complications and lower mortality rates. In these trials, patients treated with stent retriever demonstrated improved rates of functional independence. While in MR CLEAN only 82% cases in IAT patients were treated with stent retrievers and functional independence was achieved in 33% of cases, in SWIFT PRIME [16], EXTEND-IA [17], and REVASCAT [18] all patients in the IAT groups received stent retriever exhibiting functional independence rates from 43.7 to 71%. An 2016 meta-analysis reported a pooled computed tomography angiography (CTA) recanalization rate of 83% (95% CI, 73–91%) and 90-day functional independence rate of 46% (95% CI, 35–58%) across nine studies [10]. The DAWN trial in 2018 extended the therapeutic window for IAT, demonstrating optimized functional outcomes in patients treated up to 24 h after symptom onset [19]. This extended treatment window has been corroborated by several additional studies [20–22].

To facilitate risk stratification and patient selection for endovascular therapy, the Totaled Health Risks in Vascular Events (THRIVE) score offers a robust tool for evaluating outcomes in acute ischemic stroke following urgent thrombolytic revascularization [23]. The THRIVE score comprises five components, including age, the National Institutes of Health Stroke Scale (NIHSS), and the presence of atrial fibrillation, diabetes mellitus, and hypertension. Numerous Studies [24–26] have demonstrated an association between the THRIVE score and 90-day functional outcomes, measured by the modified Rankin score (mRS). Moreover, the THRIVE score has been successfully applied in various clinical contexts, including: the patients with posterior circulation occlusion [27], prediction of events of hemorrhagic transformation after revascularization therapy [28], and mortality prediction [29]. Recent evidence suggests that THRIVE score may also be valuable in predicting functional outcomes following IAT [24, 27, 30].

Immediate recanalization following endovascular thrombectomy is commonly evaluated using the Thrombolysis in Cerebral Infarction (TICI) scale [31]. A higher TICI score of 2b/3 indicates successful revascularization of the affected brain tissue, while a lower TICI score of 0–2a reflects poor recanalization after IAT. The TICI scale provides a transitional assessment immediately after endovascular therapy and is significantly associated with the post-stroke outcomes. However, few studies have examined the association between the THRIVE score and TICI grading after IAT. We propose the THRIVE score could be utilized by emergency medical services to evaluate the likelihood of successful recanalization in acute ischemic stroke patients. This study aims to evaluate whether the THRIVE score can predict immediate revascularization status following endovascular thrombectomy.

## Methods

### Study design

This retrospective cohort study aimed at evaluating the predicting ability of the THRIVE score for revascularization status and functional outcomes in acute ischemic stroke patients following intra-arterial thrombectomy therapy. This study was approved by the Joint Institutional Review Board (IRB) of Taipei Medical University (IRB numbers: N202110064) in accordance with the Declaration of Helsinki. Informed consent was waived by the IRB due to the use of anonymous and deidentified data.

### Study population and data source

The study population was retrieved from the Acute Stroke Statistics System of Taipei City (ASSST) dataset, which was managed by the Taipei City Fire Department. Given that IAT became more prevalent in Taiwan since 2016, we retrieved data from the ASSST dataset, covering the period from January 1, 2017 to May 31, 2022. The ASSST dataset was documented by the emergency medical technicians (EMTs) on scene, and by emergency physicians and neurologists after comprehensive evaluation and treatment in each of the 10 advanced emergency responsibility hospitals in Taipei city, including Taipei Veterans General Hospital, Cheng Hsin General Hospital, Shin Kong Wu Ho-Su Memorial Hospital, Tri-Service General Hospital, Mackay Memorial Hospital, National Taiwan University Hospital, Taipei City Hospital Renai Branch, Cathay General Hospital, Taipei Medical University Hospital, and Wanfang Hospital. In brief, the advanced emergency responsibility hospitals in Taiwan are required to manage various critical medical conditions. For acute ischemic stroke, these hospitals must maintain 24/7 capabilities for administering thrombolysis or performing arterial thrombectomy [32]. Patients with incomplete records of clinical profile or brain imaging for recanalization status were excluded from further analysis.

### Measurement of covariates

The demographic data include age and sex. According to grouping of age in original study [23], age is further categorized in to ≤ 59, 60 to 79, and ≥ 80 years old. The comorbidities included in the study are hypertension, diabetes mellitus, dyslipidemia, atrial fibrillation, previous ischemic stroke, coronary heart disease, and congestive heart failure. Lifestyle risk factors of alcohol consumption and smoking were included. The severity of ischemic stroke is assessed using the National Institutes of Health Stroke Scale (NIHSS) and categorized into three levels based on the original study design: mild (NIHSS ≤ 10), moderate (NIHSS 11–20), and severe (NIHSS ≥ 21) [23]. The variables involving revascularization therapy includes IAT alone or IAT plus preceding IVT (IAT + IVT), and the time frame from symptom onset to puncture of IAT (time to IAT). The THRIVE score is calculated based on age, NIHSS score, and the Chronic Disease Scale (CDS), which includes the presence of atrial fibrillation, diabetes mellitus, and hypertension. The total score is categorized into three severity levels: 0–2 (mild), 3–6 (moderate), and 7–9 (severe). To facilitate statistical analysis and clinical interpretation, we further seek to identify an optimal threshold for dichotomizing the THRIVE score.

### Outcome measures

Our primary outcome is the Treatment in Cerebral Ischemia (TICI) score [33]. TICI 0 indicates no perfusion in angiographic image distal from the occlusion site of cerebral artery; TICI 1 indicates minimal perfusion; TICI 2a showed partial reperfusion, and TICI2b shows full refilling of the vascular territory but slower than the contralateral normal site; the TICI 3 means complete reperfusion. TICI 2b and 3 are considered good revascularization and indicate good image outcome, while TICI grade 0 to 2a showed the decreased revascularization status and means poor image outcome. Our secondary outcome is the 90 days modified Rankin scale (mRS) [34], which is a grading system to categorize patients into 6 degrees according to their daily activities. A higher score on the mRS reflects greater functional dependence in daily activities.

### Statistical analysis

In the descriptive analysis, categorical variables are presented as frequencies and percentages, while continuous variables are expressed as means with standard deviations. The Chi square test or Fisher’s Exact Test is adopted to compare the categorical variables between different outcomes, while Student t test is employed to compare the continuous variables. Logistic regression model is used to evaluate odds ratio (OR) of optimized vascular reperfusion and independent functional outcome. For primary outcome, the dependent variables are categorized into TICI scores of 2b or 3, and TICI scores of 0 to 2a. The secondary outcomes were defined by modified Rankin Scale (mRS) scores of 0 to 1 and scores of 2 to 5. Independent variables included age, sex, hypertension, diabetes mellitus, atrial fibrillation, baseline NIHSS, THRIVE score, time interval from symptom onset to initiation of IAT, management by IAT alone or IAT preceded by IVT. In multivariable logistic regression, the enter method was applied, with covariates selected based on a univariate p value < 0.05. The optimal threshold of the THRIVE score for predicting successful revascularization was determined using the maximum value of Youden’s index (sensitivity + specificity – 1). Receiver operating characteristic (ROC) curves were constructed for each variable to assess their predictive performance for favorable functional outcomes, defined as a modified Rankin Scale (mRS) score of 0–1. A subgroup analysis was performed to examine gender-based differences. Statistical analyses are conducted using SPSS software (IBM Statistics for Windows, Version 22.0. Armonk, NY: IBM Corp). A p value of < 0.05 is considered statistically significant.

## Results

### Characteristics of study population

**Fig. 1:**
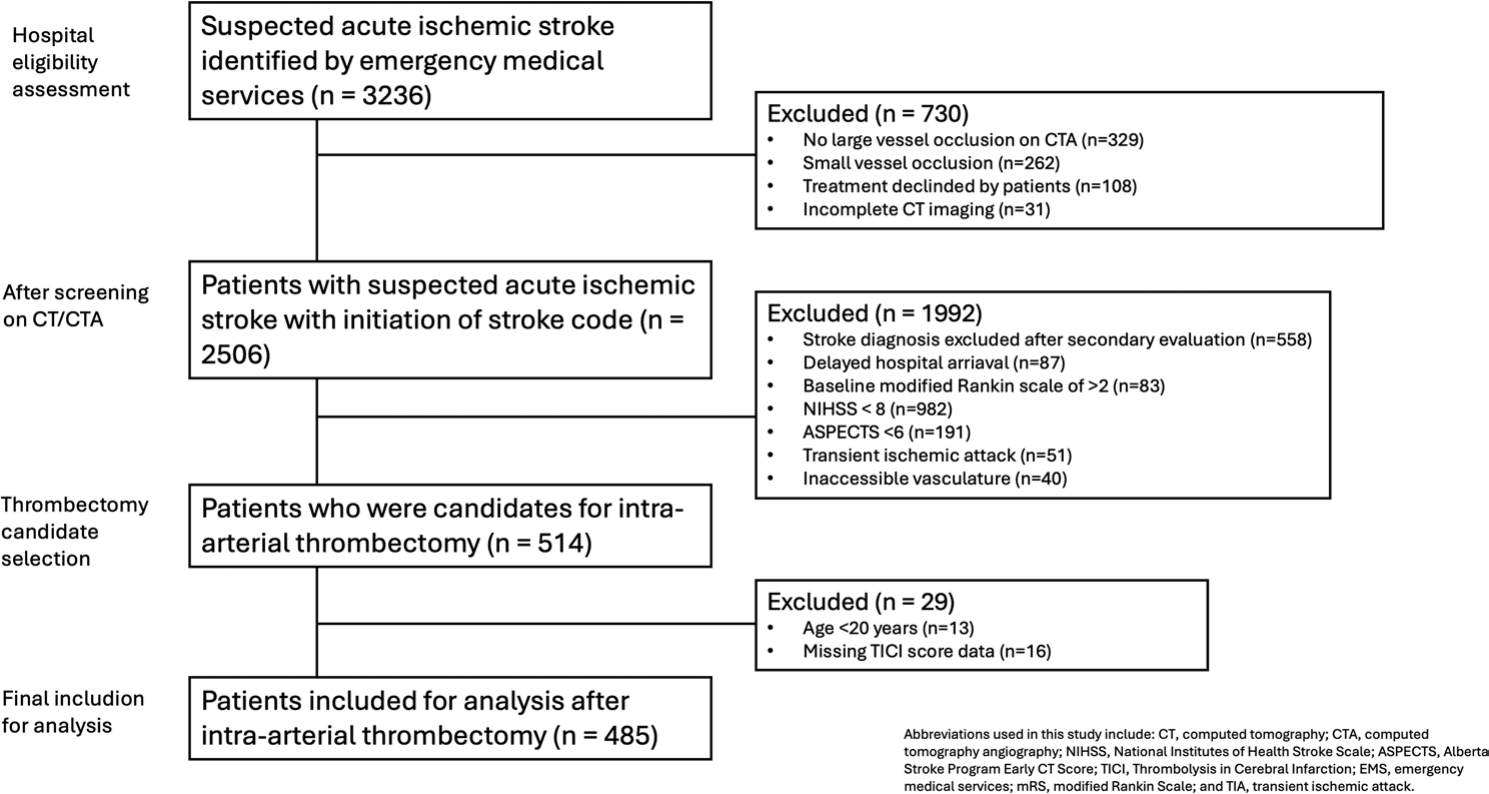
Flow diagram of the study. Flow diagram illustrating the inclusion and exclusion process of study participants. The chart outlines the number of patients assessed for eligibility, reasons for exclusion, and the final cohort included in the analysis

**Fig. 2:**
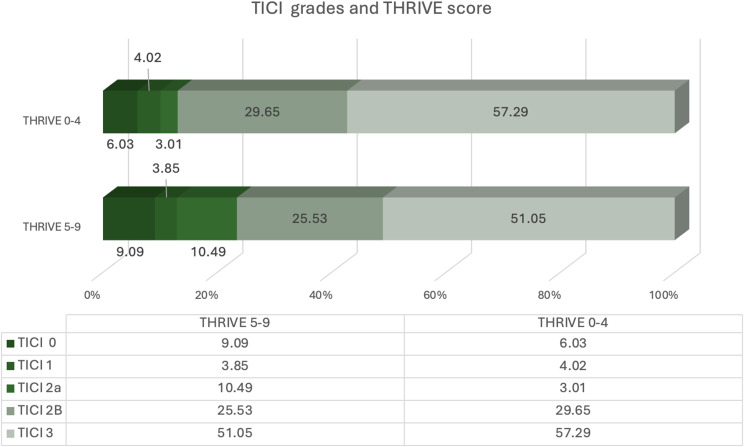
Distribution of TICI grades stratified by THRIVE score groups. The figure compares the distribution of final TICI scores between patients with low (THRIVE 0–4) and high (THRIVE 5–9) baseline THRIVE scores. Patients with lower THRIVE scores had a higher proportion of complete reperfusion (TICI 3: 57.3% vs. 51.1%) and lower rates of unsuccessful revascularization (TICI 0–2a: 13.1% vs. 23.4%) compared to those with higher THRIVE scores

From January 1, 2017, to May 30, 2022, 3236 patients were identified by EMS with suspected acute ischemic stroke. After stepwise exclusions based on imaging, clinical criteria, and data completeness, 485 patients treated with IAT were included in the final analysis. The flow diagram is shown (Fig. 1). The median age was 75.0 years (± 12.3), and 229 patients (47.2%) were women. Successful revascularization, defined as a TICI score of 2b–3, was achieved in 392 patients (80.8%), while 93 patients (19.2%) had poor revascularization outcomes (TICI 0–2a). The distribution of THRIVE scores is shown (Supplemental Fig. 1), with stratification by final TICI grades presented (Fig. 2). Higher THRIVE scores were associated with poorer angiographic outcomes, including a reduced likelihood of achieving complete reperfusion (TICI 3). A comparison of baseline characteristics between the successful and poor revascularization groups is shown in Table 1. Patients in the successful revascularization group were more likely to be male (55.6% vs. 40.9%, *p* = 0.010), receive combined intravenous thrombolysis and thrombectomy (49.7% vs. 36.6%, *p* = 0.022), and had shorter median time-to-IAT initiation (160.9 min vs. 177.0 min, *p* = 0.035). They also presented with lower THRIVE scores upon arrival at the emergency department (mean 4.8 vs. 5.3, *p* = 0.014). The majority of occlusions involved the anterior circulation (97.2% vs. 96.8%, *p* = 0.411), with only a small proportion affecting the posterior circulation or both territories. Middle cerebral artery occlusions were the most common (87.5% vs. 84.9%, *p* = 0.510), including M1 (54.3% vs. 51.6%, *p* = 0.636) and M2 segments (26.5% vs. 29.0%, *p* = 0.625). Internal carotid artery involvement was noted in 21.4% vs. 26.9% of cases (*p* = 0.257). Posterior circulation segments were infrequently involved.

**Table 1:** Population characteristics (*N* = 485)

Characteristics	TICI score of 0-2a (*N* = 93)	TICI score of 2b-3 (*N* = 392)	*P* value
Age (years)	77.01 ± 13.0	74.56 ± 12.1	0.084
Age groups, % (*N* /total N)			0.219
< 60 years	6.45% (6/93)	11.99% (47/392)	
60–79 years	47.31% (44/93)	48.72% (191/392)	
≥ 80 years	46.24% (43/93)	39.29% (154/392)	
Female, %	59.14% (55/93)	44.39% (174/392)	0.010*
Medical history, % (*N* /total N)			
Hypertension	68.82% (64/93)	60.46% (237/392)	0.135
Diabetes mellitus	27.96% (26/93)	22.96% (90/392)	0.310
Hyperlipidemia	19.35% (18/93)	22.45% (88/392)	0.516
Atrial fibrillation	45.16% (42/93)	41.84% (164/392)	0.560
Old stroke	9.68% (9/93)	17.6% (69/392)	0.061
Coronary artery disease	29.0% (27/93)	22.7% (89/392)	0.198
Congestive heart failure	6.5% (6/93)	3.1% (12/392)	0.120
Alcohol consumption	9.68% (9/93)	4.85% (19/392)	0.073
Smoking, % (*N* /total N)			0.362
nonsmoker	90.32% (84/93)	85.46% (335/392)	
quit-smoking	4.30% (4/93)	4.85% (19/392)	
smoker	5.38% (5/93)	9.69% (38/392)	
NIHSS on arrival	17.77 ± 5.5	17.53 ± 5.8	0.716
Stroke severity†, % (*N* /total N)			0.484
Mild (NIHSS of ≤ 10)	12.90% (12/93)	15.56% (61/392)	
Moderate (NIHSS of 11–20)	51.61% (48/93)	55.10% (216/392)	
High (NIHSS of ≥ 21)	35.48% (33/93)	29.34% (115/392)	
IAT + IVT (vs. IAT only)	36.6 (34/93)	49.7% (195/392)	0.022*
Stroke Territory, % (*N* /total N)			0.411
Anterior circulation	96.8% (90/93)	97.2% (381/392)	
Posterior circulation	1.1% (1/93)	2.0% (8/392)	
Anterior and posterior circulation	2.2% (2/93)	0.8% (3/392)	
Occlusion Segment, % (*N* /total N)			
MCA (M1, M2, and others)	84.9% (79/93)	87.5% (343/392)	0.510
M1	51.6% (48/93)	54.3% (213/392)	0.636
M2	29.0% (27/93)	26.5% (104/392)	0.625
ACA	2.2% (2/93)	5.9% (23/392)	0.145
ICA	26.9% (25/93)	21.4% (84/93)	0.257
PCA	0% (0/93)	0.4% (2/93)	1.000
VA	2.2% (2/93)	0.0% (0/93)	0.036*
BA	1.1% (1/93)	2.0% (8/93)	1.000
Time to IAT (min)	177.0 ± 70.3	160.9 ± 63.2	0.035*
THRIVE score (mean, median)	5.27 ± 1.56	4.80 ± 1.89	0.014*
Groups of THRIVE score, % (*N* /total N)			0.038*
0–2	3.23% (3/93)	10.97% (43/392)	
3–6	73.12% (68/93)	68.11% (267/392)	
7–9	23.66% (22/93)	20.92% (82/392)	

Abbreviations: ACA, anterior cerebral artery; BA, basilar artery; ICA, internal carotid artery; IVT, intra-venous thrombolysis; MCA, middle cerebral artery; TICI, Thrombolysis in Cerebral Infarction; NIHSS, National Institutes of Health Stroke Scale; THRIVE, Totaled Health Risks in Vascular Events; IAT, intra-arterial thrombectomy; VA, vertebral artery. Continuous variables are expressed as the mean ± standard deviation; categoric variables are expressed as the percentage and case number. *Statistically significant at *p* < 0.05. †The cut-off interval for baseline NIHSS was in accordance with THRIVE score classification

### Univariate predictors of successful revascularization

In univariate logistic regression analyses (Table 2), male sex (OR, 1.81; 95% CI, 1.15–2.87; *p* = 0.011), combined administration of IVT and IAT (OR, 1.72; 95% CI, 1.08–2.74; *p* = 0.023), and lower THRIVE scores (OR per point increase, 0.88; 95% CI, 0.77–0.99; *p* = 0.028) were significantly associated with higher odds of successful revascularization. Time from symptom onset to IAT initiation showed a borderline association (OR, 1.00; 95% CI, 0.98–1.00, *p* = 0.038).

**Table 2:** Predictors of Audio Volume Up favorable outcomes of thrombolysis in cerebral infarction in 485 patients: univariate and multivariate analyses

Characteristics	Univariate analysis		Multivariate analysis Model 1		Multivariate analysis Model 2	*P* value
	OR (95% CI)	*P* value	OR (95% CI)	*P* value	OR (95% CI)	
Age (continuous)	0.98 (0.96–1.00)	0.09				
≤ 59 years	(reference)	-				
60 to 79 years	0.55 (0.22–1.38)	0.20				
≥80 years	0.46 (0.48–1.14)	0.09				
Sex (male vs. female)	1.81 (1.15–2.87)	0.01*	1.88 (1.16–3.07)	0.01*	1.91 (1.17–3.30)	0.01*
IAT + IVT vs. IAT only	1.72 (1.08–2.74)	0.02*	1.69 (1.03–2.76)	0.04*	1.69 (1.03–2.77)	0.04*
Time to IAT (minute)	1.00 (0.98-1.00)	0.04*	0.99 (0.98–0.99)	0.02*	0.99 (0.98–0.99)	0.02*
THRIVE score						
0–2	(reference)	-				
3–6	0.27 (0.08–0.90)	0.03*				
7–9	0.27 (0.08–0.93)	0.04*				
THRIVE score (continuous)	0.87 (0.77–0.99)	0.03*			0.89 (0.78–1.02)	0.08
THRIVE score (< 5 vs. ≥ 5)	2.04 (1.23–3.03)	0.01*	1.91 (1.13–3.24)	0.02*		

Abbreviations: TICI, Thrombolysis in Cerebral Infarction; NIHSS, National Institutes of Health Stroke Scale; THRIVE, Totaled Health Risks in Vascular Events; IAT, intra-arterial thrombectomy; IVT, intra-venous thrombolysis. CI indicates confidence interval; OR, odds ratio. *Statistically significant at *p* < 0.05

### Categorical THRIVE score and optimal cut-off

When categorized by severity (Table 2), patients with mild THRIVE scores (0–2) had significantly greater odds of complete revascularization compared with those with moderate (3–6) or severe (7–9) scores (OR for moderate vs. mild, 0.27; 95% CI, 0.08–0.90; *p* = 0.034; OR for severe vs. mild, 0.27; 95% CI, 0.08–0.93; *p* = 0.037). These findings underscore the inverse relationship between THRIVE score burden and the likelihood of successful endovascular reperfusion.

**Fig. 3:**
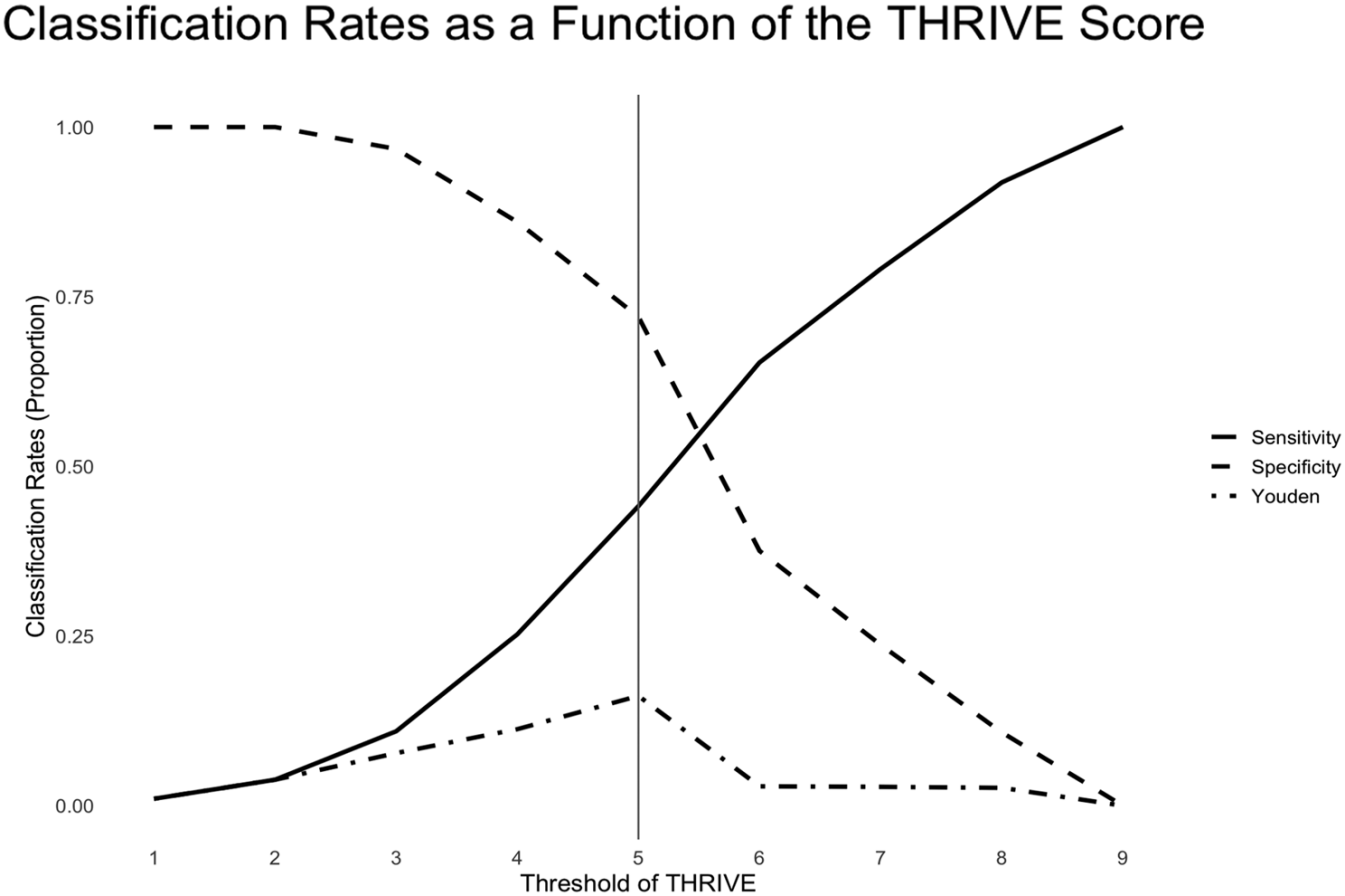
Optimized threshold of THRIVE score for predicting successful revascularization (TICI 2b–3). Sensitivity, specificity, and Youden’s index are plotted as functions of THRIVE score thresholds. The optimal cutoff of 5.0 maximized Youden’s index, indicating the best trade-off between sensitivity and specificity for predicting TICI 2b–3 reperfusion

To identify the most clinically informative threshold, we conducted a diagnostic accuracy analysis across THRIVE score cut-offs ranging from 1 to 9 (Supplemental Table 1). Based on Youden’s index, a THRIVE score of 5 was identified as the optimal cut-off (Fig. 3). Among patients with THRIVE < 5, 86.9% achieved successful recanalization, compared to 76.6% with THRIVE ≥ 5. Dichotomized analysis confirmed that a THRIVE score < 5 was associated with significantly higher odds of successful revascularization (OR, 2.04; 95% CI, 1.23–3.03; *p* = 0.005).

### Multivariable predictors of successful revascularization

In multivariable logistic regression, a THRIVE score < 5 remained an independent predictor of successful revascularization (adjusted OR, 1.91; 95% CI, 1.13–3.24; *p* = 0.016; Table 2). Modeled as a continuous variable, however, the THRIVE score did not retain statistical significance. Additional independent predictors included male sex, use of combined IVT and IAT, and shorter time to IAT initiation. Among the examined variables, ROC analysis (Supplemental Fig. 2) showed that the dichotomous THRIVE score had the best discriminatory ability for predicting successful revascularization (TICI 2b–3), with an AUC of 0.581 (95% CI, 0.517–0.645; *p* = 0.018). Although statistically significant, this represents only modest discriminatory power.

### Combination of THRIVE and TICI for predicting functional outcome

**Fig. 4:**
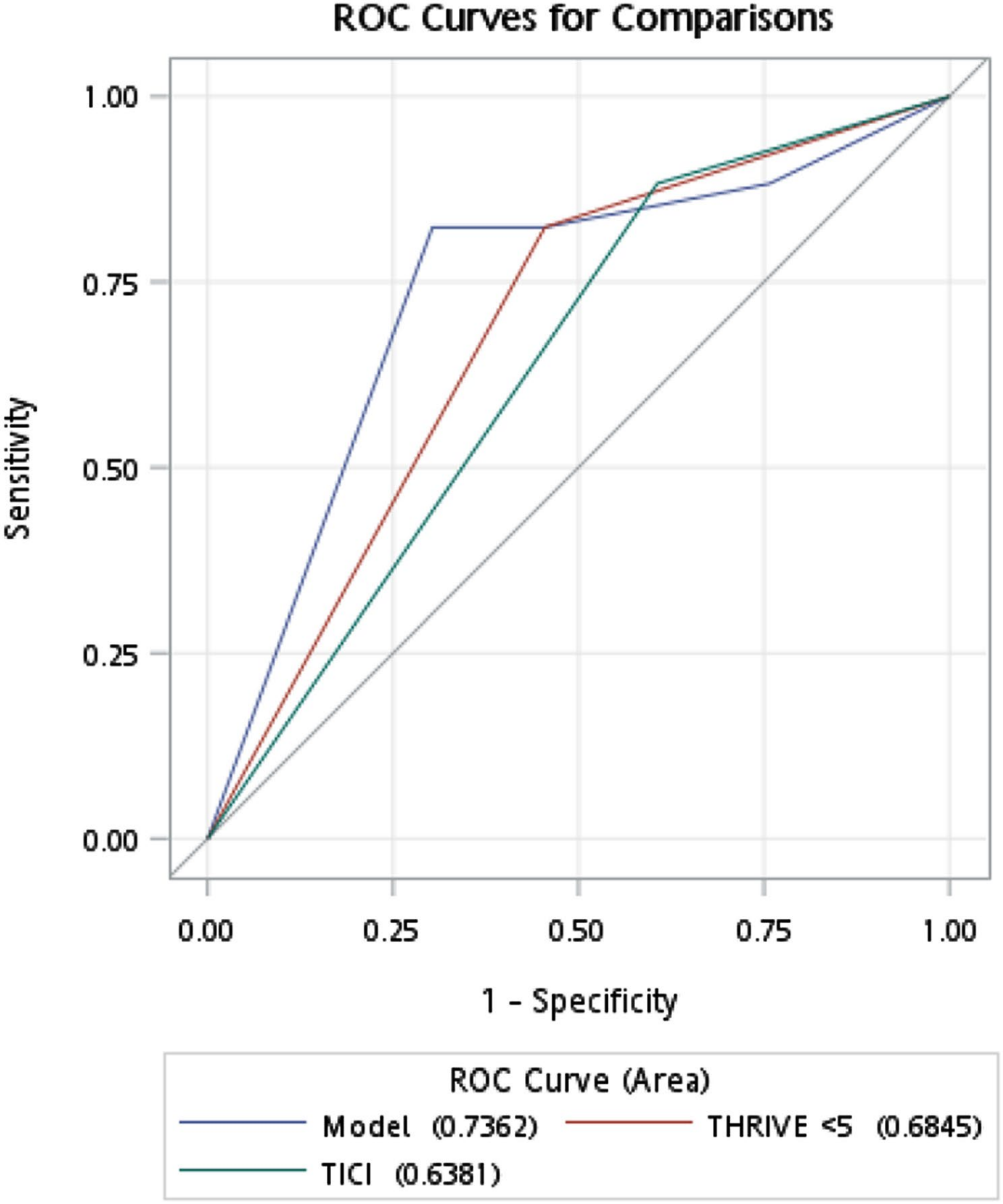
Receiver of operating characteristic curve of THRIVE score combined with TICI grade for predicting 3-month modified Rankin Scale (mRS) in patients treated at TMUH. The figure compares the predictive performance of THRIVE score < 5, final TICI grade, and a combined model in relation to 90-day functional independence. The area under the curve (AUC) was 0.7362 for the combined model, 0.6845 for THRIVE < 5 alone, and 0.6381 for TICI alone

The combination of THRIVE score and final TICI grade demonstrated moderate-to-good predictive performance for 90-day functional independence, with an area under the curve (AUC) of 0.74 (Fig. 4). Additionally, a moderate inverse association was observed between TICI score and 90-day mRS (Spearman’s ρ = − 0.38; 95% CI, − 0.63 to − 0.11).

### Age stratification analysis

We conducted a stratified analysis using 77 years as the cutoff, which represented the median age in our cohort (Supplemental Table 2). Among patients aged < 77 years (*n* = 241), both continuous and dichotomized THRIVE scores (< 5 vs. ≥5) were significantly associated with successful revascularization (adjusted OR for continuous score, 0.71; 95% CI, 0.57–0.88; *p* = 0.002; for dichotomized score, 3.05 95% CI, 1.51–6.16; *p* = 0.002). In contrast, no significant associations were observed in the ≥ 77-year group (*n* = 244). Additional subgroup comparisons (Supplemental Table 3) revealed that younger patients had significantly lower mean THRIVE scores (4.1 vs. 5.7, *p* < 0.001), lower comorbidity burden as measured by the Chronic Disease Score (CDS), and milder stroke severity based on NIHSS categories (all *p* < 0.01). These findings suggest that the association between the THRIVE score and revascularization success is stronger among younger patients.

### Sex stratification analysis

In our cohort, female patients were older than male patients (mean age 77.21 ± 12.86 vs. 73.00 ± 11.47 years) and had a higher proportion aged ≥ 80 years (50.7% vs. 31.6%), contributing to higher THRIVE scores among women (Supplemental Fig. 3; Supplemental Table 4). Apart from age, there were no significant differences between sexes in comorbidities, initial NIHSS scores, type of revascularization therapy, or time from symptom onset to IAT initiation (Table 3). A THRIVE score < 5 was significantly associated with successful revascularization in women (OR, 2.70; 95% CI, 1.30–5.55; *p* = 0.008), but not in men (OR, 1.37; 95% CI, 0.68–2.77; *p* = 0.376). Among male patients, predictors of reperfusion included combined IVT and IAT (OR, 2.24; *p* = 0.035) and shorter door-to-puncture time (OR, 0.99 per minute; *p* = 0.003).

**Table 3:** Subgroups analysis: predictors of higher TICI of thrombolysis in cerebral infarction in patients of different genders: univariate analyses for successful revascularization

Characteristics	Male (*n* = 256)		Female (*n* = 229)	
	OR (95% CI)	*P* value	OR (95% CI)	*P* value
Age (continuous)	0.98 (0.95–1.01)	0.15	0.99(0.97–1.02)	0.61
Age (categorical)				
≤ 59 years	(reference)	0.18	(reference)	0.72
60 to 79 years	6.31 (0.79–50.30)	0.08	0.98(0.32–2.94)	0.97
≥ 80 years	0.93 (0.45–1.95)	0.85	1.29(0.68–2.46)	0.44
Hypertension	1.02 (0.50–2.07)	0.96	0.51(0.26-1.00)	0.05
Diabetes Mellitus	1.24 (0.53–2.86)	0.62	0.54(0.27–1.05)	0.07
Atrial fibrillation	0.67 (0.34–1.34)	0.26	1.15(0.63–2.12)	0.65
Dyslipidemia, (yes vs. no)	2.21 (0.75–6.55)	0.15	0.96(0.48–1.93)	0.91
IAT + IVT (vs. IAT only)	2.24 (1.06–4.74)	0.04*	1.53(0.82–2.82)	0.177
Door to IAT (min)	0.99 (0.98–0.99)	0.003*	1.00(0.99-1.00)	0.88
Arrival NIHSS (continuous)	0.99 (0.93–1.05)	0.74	0.998(0.94–1.05)	0.93
THRIVE score (continuous)	0.88 (0.73–1.06)	0.166	0.89(0.75–1.05)	0.173
THRIVE score (< 5 vs. ≥ 5)	1.37 (0.68–2.77)	0.376	2.70 (1.30–5.55)	0.008*

Abbreviations: TICI, Thrombolysis in Cerebral Infarction; NIHSS, National Institutes of Health Stroke Scale; THRIVE, Totaled Health Risks in Vascular Events; IAT, intra-arterial thrombectomy; IVT, intra-venous thrombolysis. CI indicates confidence interval; OR, odds ratio; *Statistical significance was defined as p value < 0.05

## Discussion

This study demonstrates that the THRIVE score is a reliable and clinically relevant predictor of angiographic revascularization success in patients undergoing IAT for acute ischemic stroke. A THRIVE score < 5 was consistently associated with higher odds of successful reperfusion and, when paired with final TICI grading, enhanced the prediction of functional independence at 90 days. This association was particularly pronounced in female patients, whose higher THRIVE scores—driven largely by older age—may have strengthened the score’s discriminative performance.

### Expanding the role of the THRIVE score in acute stroke care

While the THRIVE score has been widely validated for predicting 90-day functional outcomes after IAT, its role in forecasting immediate angiographic success has remained largely unexplored [35, 36]. In this study, we found that a THRIVE score < 5 was not only associated with favorable long-term outcomes but also with higher odds of successful revascularization—extending the score’s clinical utility to procedural prediction. Prior research, including work by Rajan et al. [37], has demonstrated that TICI grade—a dynamic procedural marker—outperforms baseline imaging scores such as ASPECTS in forecasting functional recovery. Moreover, even patients with low ASPECTS may experience favorable outcomes if full reperfusion is achieved [38]. By showing that the THRIVE score correlates with both revascularization success (Table 2)— demonstrating the highest discriminatory performance among key clinical variables (supplemental Fig. 2)—and long-term independence, our findings support its integration into pre-procedural risk stratification and post-procedural prognostication in endovascular stroke care.

### Age as a dominant risk modifier

Age remains the most heavily weighted component of the THRIVE score, and its influence was clearly reflected in our cohort. Older patients were less likely to achieve successful revascularization and more likely to exhibit poor functional outcomes—findings that are well aligned with prior registry-based data [39–41]. Notably, women in our cohort were significantly older than men and had a lower rate of successful revascularization. These age-related disparities may partially account for the observed sex-specific performance of the THRIVE score. Data from large-scale registries such as Get With The Guidelines–Stroke have consistently shown that advanced age is associated with higher NIHSS scores, increased comorbidity burden, and worse post-stroke recovery. Moreover, older adults are more likely to experience treatment delays or receive less aggressive therapy, which may further limit the effectiveness of reperfusion strategies [40].

### Impact of vascular comorbidities

Interestingly, the individual comorbidities integrated into the THRIVE score—atrial fibrillation, hypertension, and diabetes mellitus—did not independently predict revascularization success or 90-day outcomes in our analysis. This observation aligns with recent IAT-specific prognostic models, which have similarly excluded these variables as independent predictors [42–44]. One plausible explanation lies in the synergistic nature of vascular risk factors, where the aggregate burden may carry more prognostic weight than any single comorbidity. This principle is well illustrated by the Charlson Comorbidity Index [45], which emphasizes cumulative risk. Prior studies have shown that multimorbidity—particularly the presence of two or more chronic conditions—substantially increases the risk of post-stroke mortality and functional dependence [46, 47].

### Sex-specific disparities in revascularization and prognosis

In our sex-stratified analysis, a THRIVE score < 5 was significantly associated with successful revascularization in women but not in men, reflecting underlying differences in baseline risk profiles. However, this observation should be interpreted cautiously. A difference in subgroup outcomes does not, by itself, constitute evidence of a statistically significant interaction by sex. We hypothesize that the observed disparity may reflect differences in age distribution and baseline risk profiles between men and women in our cohort. Women in our cohort were older, had higher THRIVE scores, and exhibited lower reperfusion rates—consistent with prior studies linking age to reduced treatment eligibility and poorer outcomes [39–41, 48–50]. Older women are more likely to experience functional limitations and barriers to care, which may further attenuate the benefits of acute intervention [40, 51]. In addition, sex-specific vascular risks contribute to this disparity: comorbidities such as hypertension and diabetes confer a disproportionately higher stroke risk in women [52–54], as shown in the UK Biobank cohort [55].

Several biological and clinical factors may explain the higher likelihood of successful revascularization observed in male patients. Anatomically, men generally have larger arterial vessel diameters, which may facilitate catheter access and device maneuverability during mechanical thrombectomy, thereby improving procedural success [56]. Thrombi in male patients also tend to be more enriched in red blood cells (RBCs), resulting in softer clots that are generally easier to retrieve and fragment [57]. In contrast, thrombi in female patients are typically richer in fibrin and white blood cells, producing denser, more organized structures that may reduce the effectiveness of first-pass thrombectomy attempts and limit responsiveness to thrombolytic therapy [58, 59]. These mechanistic explanations are, however, speculative. Our study did not directly evaluate clot composition or vascular anatomy. We therefore present these points as hypothesis-generating and suggest that future studies explore these potential mechanisms in larger, diverse cohorts.

Differences in stroke etiology may further contribute to this sex disparity. Men exhibit a higher prevalence of LVO and a lower incidence of cardioembolic stroke compared to women [60–62]. Since mechanical thrombectomy is particularly effective in LVO, this distribution likely favors improved revascularization outcomes in male patients [63]. Additionally, molecular mechanisms such as fibrinolytic regulation may play a role. Plasminogen activator inhibitor-1 (PAI-1), a key endogenous inhibitor of tissue plasminogen activator, impairs fibrinolysis and has been associated with reduced recanalization rates [64–66]. Men have been reported to have lower circulating PAI-1 levels than women in both acute coronary syndrome and ischemic stroke settings [67–71], potentially contributing to more favorable thrombolytic responsiveness and reperfusion outcomes. Supporting this, animal models genetically modified to express lower levels of human PAI-1 have demonstrated improved cerebral reperfusion and neurological recovery following ischemic stroke, further implicating PAI-1 as a mediator of revascularization success [72, 73].

Prehospital factors compound these differences—women were more likely to live alone, suffer unwitnessed strokes, and experience treatment delays due to delayed EMS activation [74–76]. Woman also more commonly presented with atypical symptoms, including dizziness, headache, or altered mental status, which are often missed by standard screening tools like FAST [75–82]. Together, these findings underscore the multifactorial nature of sex disparities in stroke care and highlight the importance of sex-sensitive approaches to risk prediction, triage, and treatment planning.

### Clinical translation and future directions

Our findings support the broader use of the THRIVE score as a dual-purpose tool for both pre-treatment risk stratification and post-treatment outcome prediction in acute stroke care. In prehospital settings, where detailed neurological assessments are limited, THRIVE offers a practical alternative based on routinely available information such as age and vascular comorbidities. Its predictive value is further enhanced when combined with post-procedural TICI grading, yielding moderate-to-good accuracy for functional independence at 90 days (AUC 0.74). This combined approach may inform triage decisions, procedural planning, and early prognostic counseling. Future research should refine integrated models that combine clinical, imaging, and procedural data, with attention to sex-specific disparities in stroke presentation and response.

### Limitations

This study has some limitations. First, the ASSST registry includes only patients transported via EMS, excluding those who arrived by other means, which may limit generalizability. However, all diagnoses were confirmed through neuroimaging, enhancing diagnostic specificity compared to administrative datasets. Second, the retrospective design introduces potential selection bias, particularly in cases where treatment decisions were influenced by patients or caregivers. Third, although a THRIVE score < 5 emerged as an independent predictor of successful revascularization, its discriminatory performance (AUC 0.581) should be interpreted as modest rather than strong. This suggests that the THRIVE score offers useful —but limited—predictive capability in this context. Accordingly, its clinical application should be viewed as complementary to, rather than a substitute for, other clinical and imaging-based predictors. We acknowledge this limitation and recommend that future studies investigate refined models integrating THRIVE with imaging and procedural markers.Despite these limitations, the association between lower THRIVE scores and successful revascularization remained robust and clinically relevant.

## Conclusion

The THRIVE score offers a pragmatic, accessible tool for predicting revascularization success prior to IAT and enhances long-term outcome prediction when combined with the TICI grade. Its simplicity makes it particularly valuable in prehospital and early hospital triage, where rapid risk stratification is critical. The score’s sex-specific prognostic performance underscores the need for tailored strategies in stroke care delivery and future risk modeling. These findings support broader integration of THRIVE-based metrics into endovascular treatment algorithms.

## Supplementary Information

Below is the link to the electronic supplementary material.

Supplementary Material 1

## Data Availability

The data used in this study were provided by the Taipei City Fire Department, Taipei, Taiwan. The application process was reviewed and approved by the Institutional Review Board of Taipei Medical University, followed by submission of the research proposal and a formal data request to the Taipei City Fire Department. During an official meeting with the department’s senior officers, it was confirmed that the study data could only be presented in aggregate form within the submitted manuscript. It cannot be shared in any version or format with other clinical or academic institutions for secondary use. Accordingly, the original dataset is not publicly available.
