# Supplementary material for: Pre-hospital THRIVE score predicts the thrombolysis in cerebral infarction outcome post endovascular thrombectomy: an emergency medical service study
Source: BMC Emerg Med. 2025 Sep 29;25:195. doi: 10.1186/s12873-025-01352-3 (PMC12482229; doi:10.1186/s12873-025-01352-3)
Supplement: Supplementary file 1 — Supplementary Material 1 [file 12873_2025_1352_MOESM1_ESM.docx]

**Lists of Supplemental Materials**

**Tables**

Supplemental Table 1. Diagnostic Performance of the THRIVE Score Thresholds for Predicting Successful Revascularization

Supplement Table 2. Age-Stratified Subgroup Analysis of the Association Between THRIVE Score and Successful Revascularization (TICI 2b–3).

Supplemental Table 3. Comparison of THRIVE Score, Comorbidity Burden, and Stroke Severity by Age Group (<77 vs. ≥77 Years)

Supplemental Table 4. Characteristics of Male and Female Participants

**Figures**
Supplemental Figure1. Distribution of THRIVE scores in 485 patients of acute ischemic stroke.

Supplemental Figure 2. Receiver operating characteristic (ROC) curve analysis

Supplemental Figure 3. Distribution of THRIVE scores among patients with ischemic stroke following intra-arterial thrombectomy (IAT).

Supplemental Table 1. Diagnostic Performance of the THRIVE Score Thresholds for Predicting Successful Revascularization

| THRIVE score | TP (n) | FP (n) | FN (n) | TN (n) | Sensitivity (%) | Specificity (%) | Youden’s index (%) |
| --- | --- | --- | --- | --- | --- | --- | --- |
| ≥1 vs. <1 | 93 | 388 | 0 | 4 | 100.0% | 1.0% | 1.0% |
| ≥2 vs. <2 | 93 | 377 | 0 | 15 | 100.0% | 3.8% | 3.8% |
| ≥3 vs. <3 | 90 | 349 | 3 | 43 | 96.8% | 11.0% | 7.7% |
| ≥4 vs. <4 | 80 | 293 | 13 | 99 | 86.0% | 25.3% | 11.3% |
| ≥5 vs. <5 | 67 | 219 | 26 | 173 | 72.0% | 44.1% | 16.2% |
| ≥6 vs. <6 | 35 | 136 | 58 | 256 | 37.6% | 65.3% | 2.9% |
| ≥7 vs. <7 | 22 | 82 | 71 | 310 | 23.7% | 79.1% | 2.7% |
| ≥8 vs. <8 | 10 | 32 | 83 | 360 | 10.8% | 91.8% | 2.6% |
| ≥9 vs. <9 | 0 | 6 | 93 | 386 | 0.0% | 100.0% | -1.5% |

Abbreviations: FP, False Positives; FN, False Negatives; n, number THRIVE, Totaled Health Risks in Vascular Events; TN: True Negatives; TP, True Positives.

Supplement Table 2. Age-Stratified Subgroup Analysis of the Association Between THRIVE Score and Successful Revascularization (TICI 2b–3).

|  |  | OR (95% CI) | P value | Adjusted OR (95% CI)† | P value |
| --- | --- | --- | --- | --- | --- |
| Age<77  (n=241) | THRIVE score (continuous) | 0.71 (0.57–0.88) | 0.002* | 0.69 (0.54–0.88) | 0.002* |
|  | THRIVE score  (<5 vs ≥5) | 3.05 (1.51–6.16) | 0.002* | 3.61 (1.65–7.93) | 0.001* |
| age ≥77  (n=244) | THRIVE score (continuous) | 1.05 (0.87–1.26) | 0.642 | 1.08 (0.89–1.31) | 0.421 |
|  | THRIVE score  (<5 vs ≥5) | 1.11 (0.53–2.34) | 0.785 | 0.90 (0.41–1.96) | 0.780 |

CI, Confidence Interval; IAT, Intra-Arterial Thrombectomy; IVT, Intravenous Thrombolysis; OR, Odds Ratio; THRIVE, Totaled Health Risks in Vascular Events; TICI, Thrombolysis in Cerebral Infarction.

*Statistical significance at P value < 0.05.

†Models were adjusted for Sex, Preceding IVT, and Time to IAT

Supplemental Table 3. Comparison of THRIVE Score, Comorbidity Burden, and Stroke Severity by Age Group (<77 vs. ≥77 Years)

|  | Age<77 | Age ≥77 | P value |
| --- | --- | --- | --- |
| THRIIVE score ≥5 | 39.8 % | 77.9 % | <0.001* |
| THRIVE score, mean | 4.1 ± 1.7 | 5.7 ± 1.7 | <0.001* |
| CDS |  |  | 0.001* |
| 0 | 71 (29.5%) | 38 (15.6%) |  |
| 1 | 77 (32.0%) | 101 (41.4%) |  |
| 2 | 74 (49.7%) | 75 (30.7%) |  |
| 3 | 19 (38.8%) | 30 (12.3%) |  |
| NIHSS |  |  | 0.001* |
| 0-10 | 40 (16.6%) | 33 (13.5%) |  |
| 11-20 | 146 (60.6%) | 118 (48.4%) |  |
| 21 and above | 55 (22.8%) | 93 (38.1%) |  |

CDS, Chronic Disease Score; NIHSS, National Institutes of Health Stroke Scale; SD, Standard Deviation; THRIVE, Totaled Health Risks in Vascular Events.

*Statistical significance at P value < 0.05.

Supplemental Table 4. Characteristics of Male and Female Participants

|  | Male (n= 256) | Female (n= 229) | P value |
| --- | --- | --- | --- |
| Age (years) | 73.00 ± 11.47 | 77.21 ± 12.86 | <0.001* |
| Age (years) |  |  |  |
| ≤ 59 | 36.7% (94/256) | 21.8% (50/229) | <0.001* |
| 60 to 79 | 31.6% (81/256) | 27.5% (63/229) |  |
| ≥80 | 31.6% (81/256) | 50.7% (116/229) |  |
| Hypertension | 60.9% (156/256) | 63.3% (145/229) | 0.06 |
| Diabetes Mellitus | 24.2% (62/256) | 23.6% (54/229) | 0.87 |
| Atrial fibrillation | 39.1% (100/256) | 46.3% (106/229) | 0.11 |
| IAT+IVT vs IAT alone | 44.9% (115/256) | 49.8% (114/229) | 0.29 |
| Arrival NIHSS | 17.41 ± 5.83 | 17.76 ± 5.56 | 0.51 |
| Door to puncture time | 143.32 65.10 | 142.71 64.23 | 0.92 |
| THRIVE score | 4.69 ± 1.87 | 5.11 ± 1.79 | 0.01* |
| THRIVE score ≥5 | 53.9% (138/256) | 64.6% (148/229) | 0.02* |

Abbreviations: TICI, Thrombolysis in Cerebral Infarction; NIHSS, National Institutes of Health Stroke Scale; THRIVE, Totaled Health Risks in Vascular Events; IAT, intra-arterial thrombectomy; IVT, intra-venous thrombolysis. Continuous variables are expressed as the mean ± standard deviation; categoric variables are expressed as the percentage and case number. *Statistically significant at p<0.05.


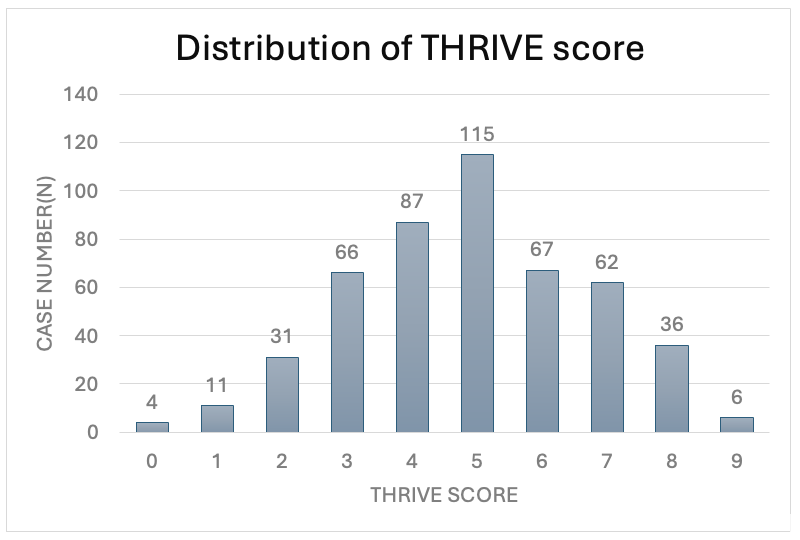


Supplemental Figure1. Distribution of THRIVE scores in 485 patients of acute ischemic stroke


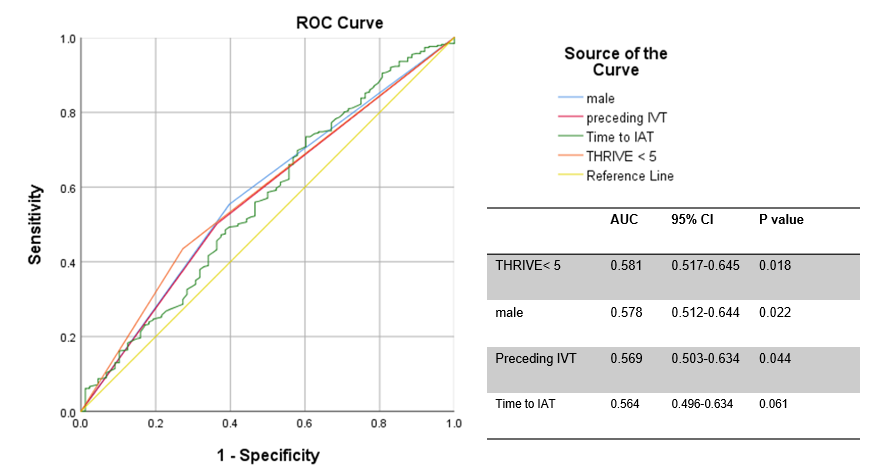


Supplemental Figure 2. Receiver operating characteristic (ROC) curve analysis illustrating the predictive performance of the Totaled Health Risks in Vascular Events (THRIVE) score, sex, preceding intravenous thrombolysis (IVT), and time to intra-arterial thrombectomy (IAT) in forecasting successful revascularization. The area under the curve (AUC) and corresponding 95% confidence intervals (CIs) are reported for each variable. *Abbreviations:* AUC, area under the curve; CI, confidence interval; IVT, intravenous thrombolysis; IAT, intra-arterial thrombectomy; THRIVE, Totaled Health Risks in Vascular Events.


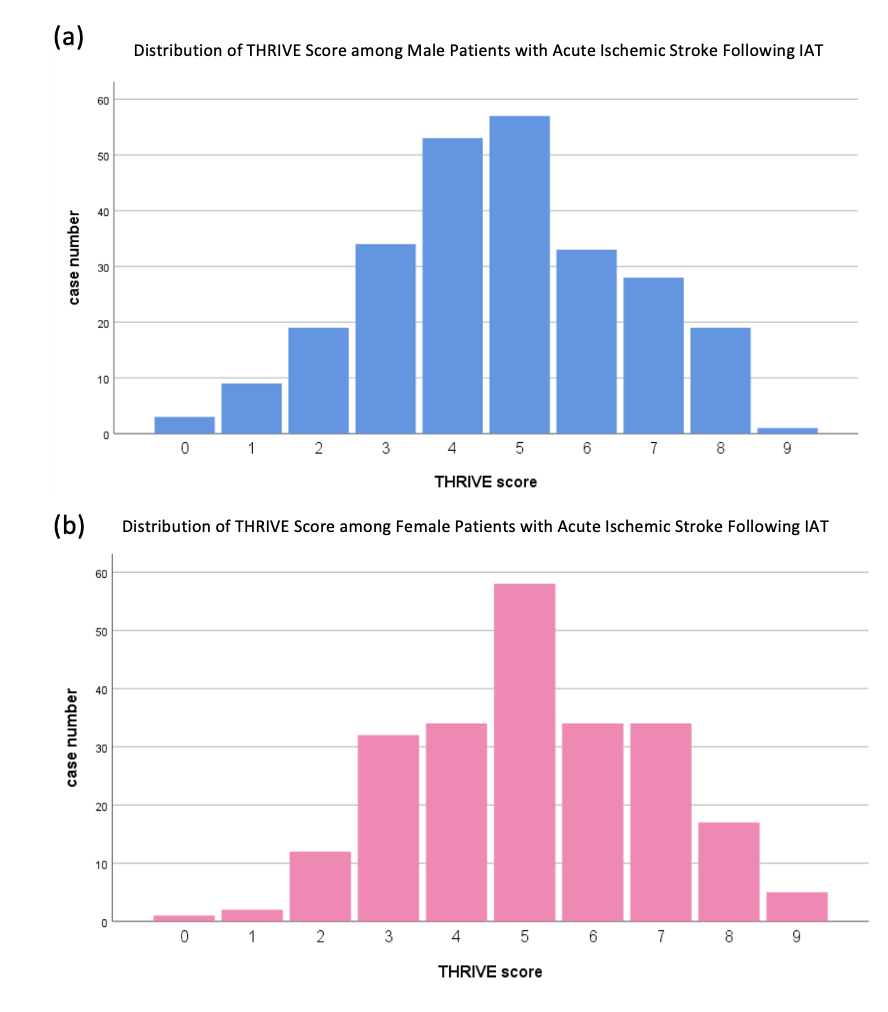


Supplemental Figure 3. Distribution of THRIVE scores among patients with ischemic stroke following intra-arterial thrombectomy (IAT). (a) Male subgroup. (b) Female subgroup
